# Safety, Pharmacokinetics, and Pharmacodynamics of the ADAMTS‐5 Inhibitor GLPG1972/S201086 in Healthy Volunteers and Participants With Osteoarthritis of the Knee or Hip

**DOI:** 10.1002/cpdd.1042

**Published:** 2021-12-02

**Authors:** Ellen van der Aar, Henri Deckx, Sonia Dupont, Ann Fieuw, Stephane Delage, Staffan Larsson, André Struglics, L. Stefan Lohmander, Agnes Lalande, Emilie Leroux, David Amantini, Paul Passier

**Affiliations:** ^1^ Galapagos NV Mechelen Belgium; ^2^ Galapagos SASU Romainville France; ^3^ Department of Clinical Sciences Lund Orthopaedics Faculty of Medicine Lund University Lund Sweden; ^4^ Institut de Recherches Internationales Servier (I.R.I.S.) Servier Suresnes France

**Keywords:** ADAMTS‐5, GLPG1972/S201086, osteoarthritis, pharmacodynamics, pharmacokinetics, phase 1, safety

## Abstract

GLPG1972/S201086 is a disintegrin and metalloproteinase with thrombospondin motif‐5 (ADAMTS‐5) inhibitor in development as an osteoarthritis disease‐modifying therapy. We report the safety, tolerability, pharmacokinetics, and pharmacodynamics (turnover of plasma/serum ARGS‐aggrecan neoepitope fragments [ARGS]) of GLPG1972 in 3 randomized, double‐blind, placebo‐controlled phase 1 trials. Study A, a first‐in‐human trial of single (≤2100 mg [fasted] and 300 mg [fed]) and multiple (≤1050 mg once daily [fed]; 14 days) ascending oral (solution) doses, investigated GLPG1972 in healthy men (N = 41; NCT02612246). Study B investigated multiple ascending oral (tablet) doses of GLPG1972 (≤300 mg once daily [fed]; 4 weeks) in male and female participants with osteoarthritis (N = 30; NCT03311009). Study C investigated single (Japanese: ≤1500 mg; White: 300 mg [fasted]) and multiple (Japanese, ≤1050 mg once daily; White, 300 mg once daily [fed]; 14 days) ascending oral (tablet) doses of GLPG1972 in healthy Japanese and White men (N = 88). The pharmacokinetic profile of GLPG1972 was similar between healthy participants and participants with osteoarthritis, with low to moderate interindividual variability. GLPG1972 was rapidly absorbed (median time to maximum concentration, 4 hours), and eliminated with a mean apparent terminal elimination half‐life of ≈10 hours. Steady state was achieved within 2 days of dosing, with minimal accumulation. Steady‐state plasma exposure after 300 mg of GLPG1972 showed no or minor differences between populations. Area under the plasma concentration–time curve (56.8‐67.6 μg · h/mL) and time to maximum concentration (4 hours) were similar between studies. Urinary excretion of GLPG1972 (24 hours) was low (<11%). Multiple dosing significantly reduced ARGS levels vs baseline at all time points for all doses vs placebo. GLPG1972 was generally well tolerated at all doses.

Osteoarthritis (OA) is a progressive joint disorder associated with joint pain, stiffness, and loss of function. It is characterized by structural changes of the joint, including a gradual degeneration of articular cartilage.^1^ Aggrecan, the major proteoglycan of extracellular matrix of articular cartilage, provides cartilage with elasticity and enables it to withstand compressive loads.^2^, ^3^ The functional ability of aggrecan decreases with age, in part owing to diminishing concentrations, but also because of proteolysis in the cartilage extracellular matrix resulting in a truncation of the core protein.^4^ The major contributors to aggrecan degradation by proteolysis are metalloproteases.^5^ Increased activity of matrix metalloprotease 3 and 13, and aggrecanase (aggrecanase‐1 and ‐2, also known as disintegrin and metalloproteinase with thrombospondin motifs‐4 [ADAMTS‐4] and ‐5 [ADAMTS‐5], respectively)^3^, ^6^ initiates aggrecan degradation that precedes cartilage loss.^7^, ^8^, ^9^ Cleavage of aggrecan at the ^392^Glu–^393^Ala bond in the interglobular domain by ADAMTS‐4 and ‐5 proteases results in the release of N‐terminal ^393^ARGS‐aggrecan neoepitope fragments (ARGS) from joint tissues into synovial fluid and blood. Increased levels of ARGS in serum, plasma, and synovial fluid may be a putative early biomarker of OA.^10^, ^11^, ^12^, ^13^, ^14^

Both ADAMTS‐4 and ‐5 contribute to the structural damage that characterizes human OA; however, ADAMTS‐5 could have the more pronounced role. Suppression of ADAMTS‐4 and ‐5 expression in human cartilage explants has been shown to reduce aggrecan degradation in cytokine‐stimulated normal cartilage.^15^ In a mouse model of OA, absence of the ADAMTS‐5 gene was associated with protection from cartilage degradation and mechanical allodynia,^16^, ^17^ while treatment with an anti–ADAMTS‐5 monoclonal antibody slowed cartilage degeneration and osteophyte growth and protected against mechanical allodynia.^18^ Inhibition of ADAMTS‐5 is, therefore, a potential approach for novel disease‐modifying OA drugs.^19^

GLPG1972/S201086, henceforth referred to as GLPG1972, is a small‐molecule inhibitor of ADAMTS‐5 being codeveloped by Galapagos and Servier, the chemical structure of which has been described by Brebion et al.^20^, ^21^ Biochemical half maximal values for inhibition (IC_50_) of human and rat ADAMTS‐5 by GLPG1972 were 19 and <23 nM, respectively, while in mouse femoral head cartilage explants stimulated by interleukin‐1α, glycosaminoglycan release was concentration‐dependently inhibited with an IC_50_ of 1.5 μM.^22^ In human articular cartilage explants stimulated with interleukin‐1β, generation of aggrecanase‐derived aggrecan fragments was dose‐dependently inhibited by GLPG1972, with a mean estimated IC_50_ <1 μM.^22^ In a meniscectomized rat model, GLPG1972 was associated with significant disease‐modifying OA drug activity, as measured by a reduction in Osteoarthritis Research Society International score, and a significant impact on several histomorphometric parameters.^22^ Furthermore, GLPG1972 significantly reduced femorotibial cartilage proteoglycan loss, cartilage damage, and subchondral bone sclerosis in a mouse model of OA (ie, destabilization of the medial meniscus).^22^

Early in vitro testing suggested that GLPG1972 is a potential cytochrome P450 (CYP) 3A4 inducer, a potential substrate of CYP3A4, and to a lesser extent of CYP2D6. Based on these in vitro results, 2 in vivo drug–drug interaction studies were conducted. Concomitant administration of the strong CYP3A4 inhibitor itraconazole resulted in a small increase in maximum observed plasma concentration (C_max_, 1.2‐fold) and a moderate increase in the area under the plasma concentration–time curve (AUC; 1.7‐fold) of GLPG1972. Concomitant administration of the strong CYP2D6 inhibitor paroxetine had no effect on C_max_ and only a small effect (1.2‐fold) on the AUC of GLPG1972. Another in vivo study with the sensitive CYP3A4 substrate midazolam showed that GLPG1972 has a weak induction potential for CYP3A4 (<30% decrease of AUC of midazolam).

Here, we report data from 3 randomized, placebo‐controlled phase 1 studies investigating the pharmacokinetics, pharmacodynamics, and safety of GLPG1972 in healthy volunteers and participants with OA.

## Methods

### Study Designs

Three randomized, double‐blind, placebo‐controlled, single‐center, phase 1 studies were conducted to investigate the pharmacokinetics, pharmacodynamics (measuring ARGS levels in plasma or serum), and safety of oral doses of GLPG1972. Study A was a first‐in‐human study in healthy male participants conducted in Belgium from October 26, 2015, to April 18, 2016 (NCT02612246). The main objectives were to evaluate the safety and tolerability of oral (solution) GLPG1972 compared with placebo when administered as single ascending doses (SADs) and multiple ascending doses (MADs) for 14 days. Objectives also included characterization of the pharmacokinetics and pharmacodynamics of GLPG1972 after single and multiple administrations, evaluation of potential interactions with CYP3A4 after repeated GLPG1972 dosing, and evaluation of the effect of food on pharmacokinetic parameters. Study B was a MAD study in male and female participants with knee and/or hip OA conducted in the United States from May 15, 2017, to October 25, 2017 (NCT03311009). The objectives were to evaluate the safety, tolerability, and pharmacokinetics and pharmacodynamics of increasing oral (tablet) daily doses of GLPG1972 for 29 days. Study C evaluated SADs and MADs of oral (tablet) GLPG1972 in healthy Japanese and White men for 14 days and was conducted in Japan between December 8, 2017, and July 23, 2018. The objectives were to evaluate the safety and tolerability of GLPG1972 vs placebo, and the pharmacokinetics and pharmacodynamics of GLPG1972 after single and repeated administration.

The studies were each conducted at 1 site (study A: Antwerp, Belgium; study B: Daytona Beach, Florida; study C: Osaka, Japan) in accordance with the Declaration of Helsinki and Good Clinical Practice guidelines. All participants provided written informed consent before enrollment, and study protocols were approved by the relevant approval boards (Commissie voor Medische Ethiek: Ziekenhuisnetwerk Antwerpen IRB, Antwerp, Belgium; Salus IRB, Austin, Texas; Independent Central Ethical Committee, Japan).

### Study Participants

Eligible participants in study A were healthy men (aged 18‐50 years) with a body mass index (BMI) of 18 to 30 kg/m^2^ and a weight of at least 50 kg. Study B participants were men or women (of nonchildbearing potential) aged 50 to 75 years, with a BMI of 18.0 to 34.9 kg/m^2^ and a diagnosis of OA (knee and/or hip) who had not received intra‐articular glucocorticoid or hyaluronan injections in the 3 months before screening or during the study. Study C participants were healthy Japanese and White men aged 20 to 45 years with a BMI of 18 to 30 kg/m^2^ and a body weight of at least 50 kg.

### Dose Administration

#### Study A (First in Human)

Participants were randomized 3:1 to receive GLPG1972 or placebo as an oral solution (150 mg/mL). The SAD phase comprised 2 alternating cohorts (A and B) planned to comprise 8 participants each (6 active treatment, 2 placebo). Cohort A sequentially received single oral doses of 60‐, 300‐, and 1050‐mg GLPG1972 when fasted and 300‐mg GLPG1972 when fed (high‐fat, high‐calorie breakfast started and finished 30 and 10 minutes before dosing, respectively; see Supplemental Information 1), or placebo. Cohort B sequentially received 150‐, 600‐, 1500‐, and 2100‐mg GLPG1972 when fasted, or placebo. GLPG1972 was administered in a solution of 150 mg/mL orally with a syringe, followed by participants drinking 240 mL of water. There was an interval of at least 3 days between administrations of 2 dose levels, during which a blinded interim analysis of safety and tolerability (and pharmacokinetics when available) was conducted before proceeding to the next dose level. The MAD phase consisted of 3 consecutive cohorts (C, D, and E; not crossover design), each planned to include 8 participants. Within a cohort, fed participants were randomized 3:1 to receive GLPG1972 (C, 300 mg; D, 600 mg; E, 1050 mg) or placebo, respectively, once daily for 14 days. On days 1 and 14, participants received a standard breakfast (not a high‐fat breakfast; see Supplemental Information 1), started and finished 30 and 10 minutes before dosing. On other days (days 2‐13), participants had an unspecified breakfast at home. Dosing was the same oral solution as noted above. Dose initiation was staggered between cohorts, allowing an interval of at least 6 days in which a blinded interim analysis of safety and tolerability was performed; a higher dose was only initiated in the next cohort when the preceding dose level had been judged as safe and well tolerated.

#### Study B (Participants With OA)

GLPG1972 was tested in a MAD study with 3 cohorts (not crossover design) of participants receiving 100, 200, or 300 mg of GLPG1972 (administered as 100‐mg oral tablets, direct compression) or matching placebo once daily in the fed state for 29 days. Dosing on days 1 and 14 was under fed conditions after a standard unspecified breakfast. Within each cohort, 10 participants were randomized 4:1 to GLPG1972 or placebo and were stratified for age (50‐64 years; 65‐75 years), with a minimum of 2 participants of each sex per age group.

#### Study C (Japanese and White Healthy Volunteers)

In the SAD phase under fasted conditions, 6 consecutive cohorts of 8 Japanese participants received single oral (tablet) GLPG1972 (50, 150, 300, 600, 1050, or 1500 mg) or matching placebo (in a 3:1 ratio), and 1 cohort of 8 White participants received a single oral dose of GLPG1972 300 mg or placebo (in a 3:1 ratio) (not crossover design). Dosing was with tablets (50 or 100 mg/tablet, wet granulation) taken with 240 mL (or 350 mL for doses of ≥600 mg) of water under fasted conditions. Fasting was overnight, and lunch and dinner were taken 4 and 10 hours after dosing, respectively. An identical composition of every meal was to be taken by each participant during inpatient stay. The cohort of White participants was treated in parallel with the 300‐mg cohort of Japanese participants. Dose escalation to the next group was decided on the basis of the safety data of the ongoing dose group and the pharmacokinetic data of the previous dose group. There was an interval of at least 1 week between dose groups to ensure that full safety and pharmacokinetic information was available. The MAD phase was started after availability of data on the safety of up to 1050 mg and pharmacokinetics of up to 600 mg in the SAD phase. The MAD phase consisted of 3 consecutive cohorts of 8 Japanese participants who received repeated doses of oral GLPG1972 (300, 600, or 1050 mg) or placebo (in a 3:1 ratio) and 1 cohort of 8 White participants who received repeated oral doses of GLPG1972 300 mg or placebo (in a 3:1 ratio), all under fed conditions for up to 14 days. GLPG1972 was administered as 50‐ and 100‐mg wet granulation tablets immediately following completion of the meal (within 10‐15 minutes). Following an overnight fasting period (no food) of at least 10 hours, a standardized breakfast was ingested within 20 minutes; identical composition of every meal taken by each participant during inpatient stay and exact time of meals and quantities consumed were recorded. Dose escalation was based on safety and pharmacokinetic data of the previous dose group.

### Pharmacokinetic Assessments

Concentrations of GLPG1972 in plasma and urine (from samples taken at different time points) were measured using liquid chromatography with tandem mass spectrometry (described in Supplemental Information 2). In study A, SAD parameters investigated included C_max_ and time to occurrence of C_max_ (t_max_); AUC and terminal elimination half‐life (t_1/2_) were also reported in the SAD phases. MAD parameters included AUC, apparent t_1/2_, calculated from (natural log [ln] 2)/λz (t_1/2,λz_), and renal clearance. Study B parameters included C_max_, t_max_, AUC over the dosing interval, AUC from time 0 to infinity, and the accumulation ratio based on AUC from time 0 to the last quantifiable level (AUC_0‐τ_), calculated as geometric mean accumulation ratio of AUC_0‐τ_ day 15/AUC_0‐τ_ day 1 (R_ac,AUC0‐τ_). Study C SAD parameters included C_max_, t_max_, and t_1/2_. MAD phase parameters included C_max_, t_max_, AUC_τ(24h)_, and t_1/2,z_ (t_1/2_ after *x* days of administration), and the accumulation ratio. In study A, the ratio of 6β‐OH‐cortisol/cortisol in urine collected on days −1 and 13 was used as a surrogate marker of interaction with CYP3A4 (analytical methodology for measurement of 6β‐OH‐cortisol and cortisol is reported in Supplemental Information 1).

### Pharmacodynamic Assessments

The pharmacodynamic response was assessed in the MAD phase of all studies by measuring levels of ARGS in plasma (study A) or serum (studies B and C). Samples were taken before dosing and at multiple time points after dosing on days 1 and 14 in studies A and C. Only predose samples were taken in study B on days 1, 3, 6, 8, 10, 15, 22, 29, 43, and 50. Available predose pharmacokinetic plasma samples from days 1, 3, 4, 5, 6, 8, 10, and 14 were also assessed for ARGS levels in study A. The AUC for the percentage reduction from baseline from 0 to 24 hours after dosing, and the percentage change from baseline based on predose samples (minimum concentration), and reduction from baseline to day 14 at 0 to 24 hours after dosing, were determined from individual effect‐time profiles. ARGS concentrations in all 3 studies were determined using an enzyme‐linked immunosorbent assay^23^ at the Biomedical Centre, Faculty of Medicine, Lund University, Sweden.

### Safety and Tolerability Assessments

In all studies, safety and tolerability were assessed by monitoring adverse events (AEs) from the date of signing of the informed consent form until the final follow‐up visit. Investigators collected AEs by observation; spontaneous, unsolicited reports from participants; and routine open questioning. Additional safety assessments included clinical laboratory safety assessments (ie, standard hematology, serum/plasma chemistry, coagulation tests, and urinalysis), vital signs, physical examination, Holter monitoring, and 12‐lead electrocardiogram (ECG).

### Statistical Analysis

In study A, the reported pharmacokinetic and pharmacodynamic data are descriptive with the following exceptions: proportionality between dose and ln‐transformed pharmacokinetic parameters (assessed using a mixed‐effects model with cohort and dose [SAD and MAD phases] and dose‐day interaction [MAD phase only]); assessment of food effect (SAD; based on ln‐transformed pharmacokinetic parameters [C_max_, AUC, and t_1/2,λz_]); and geometric mean ratios of fed vs fasted conditions. If there was a significant dose effect in this study, comparison between doses was performed using Tukey's test, except for t_max_, which was assessed using a nonparametric Kruskal‐Wallis test and, if statistically significant, Wilcoxon's rank‐sum tests with Moses 90% confidence intervals for pairwise comparisons were applied. In study B, dose proportionality was assessed as per study A, using a mixed‐effects model on ln‐transformed GLPG1972 parameters with participant as random effect and age, sex, day, dose, and dose‐day interaction as fixed effects. The age, sex, and dose effect on ln‐transformed R_ac,AUC0‐τ_ was evaluated from a mixed‐effects model with participant as random effect and age, sex, and dose as fixed effects. In the event of a significant dose effect, comparison between doses was tested as in study A. Time to reach steady state was assessed as in study A. In study C, descriptive statistics were calculated per treatment group for each pharmacokinetic parameter. For all studies, ARGS levels were expressed in terms of concentration at each time point and relative change from baseline to each time point. All statistical calculations were done using SAS version 9.4 (SAS Institute, Cary, North Carolina) and/or Phoenix WinNonlin versions 6.2, 6.4, and 8.0 (Certara, Princeton, New Jersey) software for statistical and pharmacokinetic computations.

## Results

### Participant Disposition and Demographics

In study A, 17 and 24 healthy participants were randomized in the SAD and MAD parts of the study, respectively. All participants completed the study except for 1 participant who was replaced after the second dosing period (SAD) for no longer meeting inclusion criteria on day −1 of the second dosing period. In study B, 30 participants with OA were randomized and received study drug. One participant in the 300‐mg dose group discontinued the study on day 16. In study C, 56 participants were randomized in the SAD phase (Japanese, n = 48; White, n = 8) and 32 participants were randomized in the MAD phase (Japanese, n = 24; White, n = 8). One White participant (300 mg) was lost to follow‐up at the last visit, but all remaining participants completed the SAD phase. In the MAD phase, 2 Japanese participants (both 1050 mg) discontinued the study on day 14.

Demographic characteristics of participants in the 3 studies are shown in Table S1. Participants had a median age of 30.0 years (SAD) and 42.0 years (MAD) in study A, and 61.5 years in study B. Japanese participants had a mean age of 25.5 years (SAD) and 27.2 years (MAD), and White participants had a mean age of 29.4 years (SAD) and 29.8 years (MAD) in study C. There was no overlap in age ranges between studies A and B. The median duration of OA in study B was 4.0 years (range, 0‐45). Most participants in studies A and B were White (80.0%‐94.1%).

### Pharmacokinetics of GLPG1972

**Figure 1:**
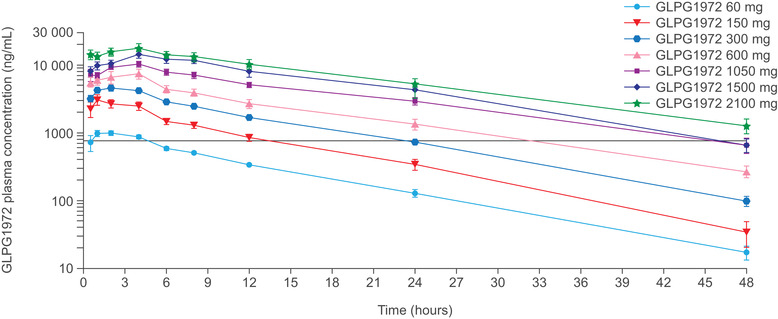
GLPG1972 plasma concentrations over time in fasted healthy participants following single ascending oral doses of GLPG1972. Data obtained from study A. Data show mean ± standard error. Black horizontal line shows shows the mean plasma concentration of the 12 hr dosing interval at steady state at the minimal efficacious dose in a rat meniscal transection model of arthritis (unpublished data).

Pharmacokinetic parameters of SAD‐phase dosing of GLPG1972 in study A are shown in Table 1A. GLPG1972 was rapidly absorbed, with a mean C_max_ of 1.1 to 18.3 μg/mL (for the dose range of 60‐2100 mg) and a mean t_max_ of 1.0 to 4.0 hours. Overall exposure increased dose proportionally between 60  and 2100 mg, whereas the increase in C_max_ was less than dose proportional (ie, 17‐fold increase in C_max_ for a 35‐fold increase in dose). Plasma concentration–time profiles after increasing single doses of GLPG1972 in healthy male participants in a fasted state are shown in Figure 1, and in the fasting vs fed state in Figure S1. The elimination phase was parallel and biphasic for all dose levels, with a mean apparent terminal elimination half‐life of 8.2 to 11.6 hours. The interindividual variability in GLPG1972 pharmacokinetic parameters was low to moderate, as shown by the coefficients of variation for C_max_ and AUC_0‐24h_. Following a single 300‐mg dose of oral (solution) GLPG1972, t_max_ was delayed in fed compared with fasted participants (2.0 vs 4.0 hours) and C_max_ was slightly lowered (4.85 vs 4.06 μg/mL). However, food had no effect on the level of overall exposure (AUC) to GLPG1972 or on its rate of elimination.

**Table 1:** GLPG1972 Plasma Pharmacokinetic Parameters in Study A

A. SAD Phase								
	GLPG1972 Dose							
			300 mg					
Pharmacokinetic Parameter	60 mg (Fasted) (n = 6)	150 mg (Fasted) (n = 6)	(Fasted) (n = 6)	(Fed) (n = 6)	600 mg (Fasted) (n = 6)	1050 mg (Fasted) (n = 6)	1500 mg (Fasted) (n = 6)	2100 mg (Fasted) (n = 6)
C_max_, μg/mL, mean (CV%)	1.05 (18.3)	3.30 (40.5)	4.85 (15.9)	4.06 (13.2)	7.91 (37.5)	10.8 (19.7)	14.9 (28.9)	18.3 (30.1)
t_max_, h, median (range)	2.0 (1.0‐4.0)	1.0 (1.0‐4.0)	2.0 (1.0‐4.0)	4.0 (4.0‐6.0)	2.5 (0.5‐4.0)	3.0 (0.5‐4.0)	4.0 (4.0‐6.0)	4.0 (0.5‐8.0)
AUC_0‐∞_, μg · h/mL, mean (CV%)	11.6 (12.9)	30.8 (31.0)^a^	58.4 (4.01)	59.4 (8.14)	100 (33.6)	184 (24.4)	234 (24.8)^a^	342 (36.5)
t_1/2,λz_, h, mean (CV%)	8.16 (13.1)	8.98 (31.2)	8.63 (13.3)	8.88 (19.9)	10.8 (24.8)	11.6 (22.8)	10.2 (19.6)^a^	11.5 (23.0)

B. MAD Phase								
	GLPG1972 Dose							
	300 mg/day (Fed) (n = 6)		600 mg/day (Fed) (n = 6)		1050 mg/day (Fed) (n = 6)			
Pharmacokinetic Parameter	Day 1	Day 14	Day 1	Day 14	Day 1	Day 14		
C_max_, μg/mL, mean (CV%)	4.03 (35.6)	4.94 (30.3)	7.68 (17.0)	10.5 (26.9)	9.34 (28.1)	16.2 (36.3)		
t_max_, h, median (range)	4.0 (4.0‐6.0)	4.0 (4.0‐4.0)	4.0 (2.0‐4.0)	4.0 (4.0‐4.0)	4.0 (4.0‐6.0)	4.0 (4.0‐6.0)		
AUC_T_, μg · h/mL, mean (CV%)	48.9 (32.6)	65.8 (32.8)	83.2 (28.0)	132 (37.4)	111 (24.2)	206 (34.5)		
AUC_0‐∞_, μg · h/mL, mean (CV%)	44.2 (NC)^b^	82.8 (36.7)	74.3 (NC)^b^	189 (44.1)^a^	106 (4.46)^c^	267 (41.0)		

AUC_T_, area under the plasma concentration–time curve over the dosing interval (ie, 24 hours after dosing); AUC_0‐∞_, area under the plasma concentration–time curve from time 0 to infinity; C_max_, maximum observed plasma concentration; CV, coefficient of variation; MAD, multiple ascending doses; NC, not calculated; SAD, single ascending doses; t_1/2,λz_, apparent terminal elimination half‐life, calculated from (ln 2)/λz; t_max_, time to occurrence of C_max_.

^a^ n = 5.

^b^ n = 2.

^c^ n = 3.

Similar pharmacokinetic parameters were observed in the SAD phase of study C (Table S3). After single oral (tablet) doses, exposure to GLPG1972 (C_max_ and AUC) in fasting Japanese participants was proportional to dose from 50 to 600 mg and less than dose proportional from 600 to 1500 mg. There was low interindividual variability in terms of C_max_ and exposure (AUC) in Japanese and White participants, with low (≤30%) values for coefficient of variation at most doses in both populations. Median t_max_ occurred at 4.0 hours for all doses (except for 3.0 hours following 1500 mg), and mean terminal half‐life ranged from 9.6 to 20 hours. Pharmacokinetic parameters were similar in Japanese and White participants following a single oral (tablet) administration of GLPG1972 300 mg (Table S3).

**Figure 2:**
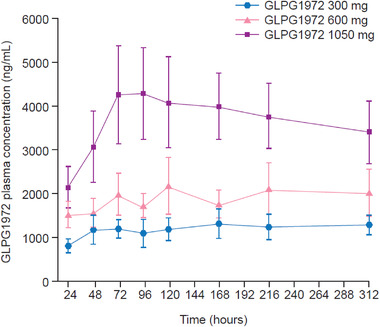
Trough GLPG1972 plasma concentrations over time in healthy participants following multiple ascending oral doses of GLPG1972. Data obtained from study A. Data show mean ± standard error.

For the MAD phase in study A, plasma exposure to oral GLPG1972 (C_max_ and AUC_T_) increased dose proportionally over the entire dose range (from 65.8 μg·h/mL at 300 mg to 206 μg·h/mL at 1050 mg, determined after 14 days of dosing; Supplementary Figure S2), while mean t_1/2,λz_ remained between 8.66 and 9.08 hours (Table 1B). Mean plasma concentrations of GLPG1972 are shown in Figure 2; steady‐state GLPG1972 plasma concentrations were reached after 2 days of dosing (day 3), with minimal accumulation over 14 days (R_ac_ of 1.3 [300 mg], 1.6 [600 mg], 1.8 [1050 mg]). On days 1 and 14, plasma t_max_ was reached by 4 hours after dosing. The elimination phase was comparable between doses on both days 1 and 14 (data not shown). Excretion of unchanged GLPG1972 in urine over a 24‐hour period was low (day 1, 6.4%; day 14, 10.6%) and no change in renal clearance of GLPG1972 was observed after repeated dosing (overall mean, 0.59 L/h). Once‐daily repeated dosing of GLPG1972 had no meaningful effect on the urinary 6β‐OH‐cortisol/cortisol ratio in 13 out of 18 participants. In the remaining 5 participants, a 2.0 to 4.7‐fold increase was observed but without apparent relationship to GLPG1972 dose or exposure. The mean urinary 6β‐OH‐cortisol/cortisol ratio is reported in Table S2.

Similar pharmacokinetic parameters to those in healthy volunteers were determined in male and female participants with OA (study B), with a mean C_max_ of 1.9 to 5.6 μg/mL (dose range, 100‐300 mg) and t_max_ reached in a median of 4.0 to 5.0 hours after dosing (Table 2), with steady state achieved in 3 to 6 dosing days. Exposure increased approximately dose proportionally with minimal accumulation (R_ac_, 1.3 [100 mg], 1.3 [200 mg], 1.4 [300 mg]) from day 1 vs day 15 or 17.

**Table 2:** GLPG1972 Plasma Pharmacokinetic Parameters in Fed Participants With OA During Multiple Dosing in Study B

	GLPG1972 100 mg (Fed)		GLPG1972 200 mg (Fed)		GLPG1972 300 mg (Fed)	
Pharmacokinetic Parameter	Day 1 (n = 8)	Day 15 (n = 8)	Day 1 (n = 8)	Day 17 (n = 8)	Day 1 (n = 8)	Day 15 (n = 7)
C_max_, μg/mL, mean (CV%)	1.90 (32.2)	2.25 (26.9)	3.44 (22.2)	3.90 (23.8)	4.83 (18.9)	5.60 (23.5)
C_τ_, μg/mL, mean (CV%)	0.351 (54.0)	0.488 (41.3)	0.698 (74.4)	1.16 (53.7)	0.807 (47.8)	1.27 (46.7)
t_max_, h, median (range)	4.0 (4.0‐5.0)	4.0 (4.0‐5.0)	5.0 (4.0‐5.0)	5.0 (4.0‐8.0)	4.0 (4.0‐8.0)	4.0 (4.0‐8.0)
AUC_0–τ_, μg · h/mL, mean (CV%)	20.0 (39.4)	24.8 (30.6)	37.7 (35.8)	49.5 (34.6)	50.7 (23.2)	67.6 (27.0)
R_ac,AUC0–τ_, mean (CV%)	NA	1.29 (17.2)	NA	1.32 (9.21)	NA	1.35 (4.84)

AUC_0–τ_, area under the plasma concentration–time curve for the dosing interval; C_τ_, plasma concentration observed at the end of the dosing interval; C_max_, maximum observed plasma concentration; CV, coefficient of variation; NA, not applicable; OA, osteoarthritis; t_max_, time to occurrence of C_max_; R_ac,AUC0–τ_, accumulation ratio based on AUC0‐τ.

R_ac_ was calculated by day 15 AUC_0–τ_ divided by day 1 AUC_0–τ_.

Similar overall MAD pharmacokinetic parameters to those described for studies A and B were determined in study C (Table 3). T_max_ occurred at 4.0 hours across all doses. Day 14 mean t_1/2_ values of 8.7 to 10 hours were found across all 3 doses (300, 600, and 1050 mg). Systemic exposure to GLPG1972 in fed Japanese volunteers was dose proportional from 300 to 1050 mg, determined at steady state (after 14 days of administration). Values for C_max_ and AUC were similar between fed Japanese and White volunteers following multiple administrations of GLPG1972 300 mg. Steady state under fed conditions was reached around day 3. Limited accumulation of GLPG1972 was observed over 14 days of repeated administration (Table S5). Consistent with the SAD phase, there was low interindividual variability of C_max_ and exposure (AUC) in the MAD phase in Japanese and White populations, with low (≤30%) values for coefficient of variation at most doses in both populations (Table S4).

**Table 3:** GLPG1972 300 mg Plasma Pharmacokinetics at Steady State in Studies A, B, and C

			Study C: MAD	
	Study A: MAD	Study B	Day 14 (n = 6), Fed	
Pharmacokinetic Parameter	Day 14 (n = 6), Fed	Day 15 (n = 7), Unknown Food Condition	Japanese	Caucasians
C_max_, μg/mL, mean (CV%)	4.94 (30.3)	5.60 (23.5)	5.56 (16)	5.08 (25)
t_max_, h, median (range)	4.0 (4.0‐4.0)	4.0 (4.0‐8.0)	4.0 (2.0‐4.0)	4.0 (4.0‐4.0)
AUC_T_, μg · h/mL, mean (CV%)	65.8 (32.8)	67.6 (27.0)	65.1 (7.4)	56.2 (27)

AUC_T_, area under the plasma concentration–time curve over the dosing interval (ie, 24 hours after dosing); C_max_, maximum observed plasma concentration; CV, coefficient of variation; MAD, multiple ascending dose; SAD, single ascending dose; t_max_, time to occurrence of C_max_.

Similar steady‐state plasma pharmacokinetic parameters were observed in the different study populations with the 300‐mg dose, which was common to all 3 studies. C_max_ was in the same range (4.94‐5.60 μg/mL) for healthy participants and those with OA, and for White and Japanese participants. Values for t_max_ (consistently 4 hours) and AUC (56.8‐67.6 μg · h/mL) were similar between studies at 300 mg.

### Pharmacodynamic Effect of GLPG1972

**Figure 3:**
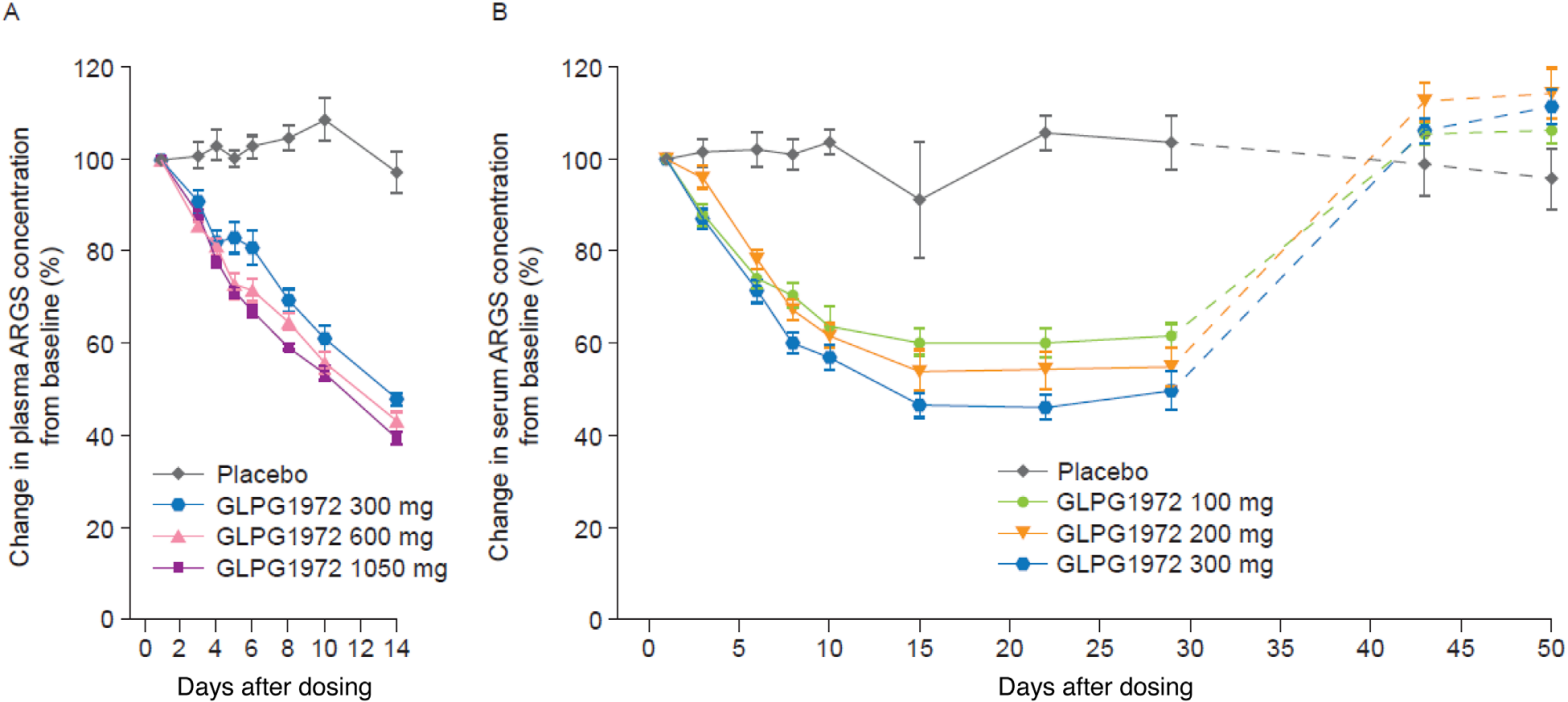
Aggrecan neoepitope ARGS concentrations at multiple time points relative to baseline based on predose samples in (A) healthy participants (plasma) and (B) participants with OA (serum). Data obtained from studies A (A) and B (B). Data show mean ± standard error. Dotted lines indicate off‐treatment periods. ARGS, ARGS‐aggrecan neoepitope fragments; OA, osteoarthritis.

In healthy volunteers (study A), once‐daily dosing for 14 days significantly reduced plasma ARGS levels vs baseline gradually over time for all doses compared with placebo (all *P* <.0001, Figure 3A); placebo was associated with no change in all studies. At day 14, reduction vs baseline in mean reduction from baseline to day 14 at 0 to 24 hours after dosing increased with increasing GLPG1972 dose (300 mg, 53.4%; 600 mg, 58.9%; 1050 mg, 61.4%; placebo, 3.2%); however, there was no statistically significant difference between doses.

In participants with OA, mean ARGS levels decreased from baseline between day 3 and day 15, followed by a floor effect between day 15 and day 29 in all GLPG1972 treatment groups (Figure 3B). On day 29, the change from baseline was 38.5% to 50.4% across GLPG1972 treatment groups (for the dose range of 100‐300 mg). Once GLPG1972 administration was stopped (after day 29), ARGS levels returned to baseline or slightly above within 14 days after cessation of dosing, remaining stable until day 50. After day 3, the percent reduction of serum ARGS levels from baseline was statistically significant in the 3 dose groups compared with placebo at all time points until the last dose (day 29), while ARGS levels remained unchanged in the placebo group throughout the study (data not shown).

In study C, no changes in serum ARGS levels were observed in placebo groups and a similar decrease of mean and individual ARGS levels was observed at day 14 with 300 mg of GLPG1972 in Japanese and White participants. For the additional doses in Japanese participants, the decrease in ARGS levels at day 14 was significant compared with placebo for all groups (*P* < .0001; data not shown).

### Safety Outcomes

Across all 3 studies, 121 participants were exposed to GLPG1972, and 34 participants were exposed to placebo. No deaths occurred in any of the studies. Across the studies, 3 serious AEs occurred in 2 participants: A Japanese participant in the 1500 mg group experienced sinus arrest and syncope (related to protocol procedures), and a White participant experienced biliary colic, all in study C. The latter event led to a temporary halt of the study, but was deemed related to the participant's medical history of cholelithiasis and not related to the study drug. Three participants experienced treatment‐emergent AEs (TEAEs) that led to treatment discontinuation. One participant receiving GLPG1972 300 mg experienced an increase in alanine aminotransferase levels on day 15 in study A (treatment was discontinued on day 16 and the TEAE resolved on day 24), and 2 participants receiving GLPG1972 1050 mg experienced several mild, nonserious TEAEs (including neck pain, pyrexia, and headache) from 10 days after dosing, which led to treatment withdrawal (all in study C).

Most TEAEs in the 3 studies were mild in severity. The most frequently reported TEAE across the studies was headache, (n = 9 participants in study A; n = 7 in study B; and n = 5 in study C).

In healthy participants, in the SAD phase of study A, 9 participants in the GLPG1972 treatment groups reported 16 TEAEs, of which 9 (headache, n = 8; dizziness, n = 1) were considered at least possibly related to treatment by the investigator. In the placebo groups, 2 participants reported 4 TEAEs, 1 of which was considered treatment related. During the MAD phase of study A, 8 participants experienced 16 TEAEs in the GLPG1972 treatment groups, 10 of which (all in the 600‐  and 1050‐mg groups) were considered possibly related to treatment by the investigator. In the combined placebo groups, 2 participants reported 5 TEAEs, 2 of which were considered treatment related.

In healthy participants in the SAD phase of study C, 13 Japanese participants (4 receiving placebo, 9 receiving GLPG1972) and 2 White participants (both receiving GLPG1972) reported TEAEs. Three patients experienced treatment‐related TEAEs, all Japanese participants receiving GLPG1972 (mild epigastric discomfort, n = 1; pyrexia, n = 2). In the MAD phase of study C, 12 participants (9 Japanese, 3 White, all receiving GLPG1972) experienced TEAEs. The most frequently reported TEAE was an increase in C‐reactive protein, reported in 5 participants (600 mg, n = 3; 1050 mg, n  =  2). Six participants (5 Japanese, 1 White, all receiving GLPG1972) experienced TEAEs that were considered treatment related by the investigator.

In participants with OA (study B), across GLPG1972 and placebo groups, 35 TEAEs were reported in 13 participants, 8 of which were considered at least possibly related to study drug. Aside from headache (5, 0, 1, and 0 participants in the 100‐, 200‐, 300‐mg, and placebo groups, respectively), each TEAE occurred in only 1 participant.

There was no relationship between the administered GLPG1972 dose and the incidence of TEAEs across all 3 studies. ECGs, vital signs, and physical examinations revealed no clinically relevant abnormalities in any study.

## Discussion

The 3 phase 1 trials reported here represent part of a clinical program designed to assess the safety and efficacy of the oral ADAMTS‐5 inhibitor GLPG1972 in humans. Overall, broadly similar pharmacokinetic profiles were observed across the populations from the 3 studies, which included healthy White and Japanese volunteers and participants with OA.

Pharmacokinetic measures showed that absorption of GLPG1972 was rapid in fasted healthy men following a single oral dose, with t_max_ in the range of 1 to 4 hours and an apparent t_1/2_ of ≈10 hours. Food slightly decreased the rate of absorption of oral (solution) GLPG1972, as might be anticipated if gastric emptying is slowed by food intake, but did not affect exposure to GLPG1972. Consistent with the t_1/2_ and once‐daily dosing regimen, steady‐state plasma concentrations were generally reached after 2 days of dosing, and there was minimal accumulation with repeat dosing, with a similar accumulation ratio reported in healthy participants and participants with OA for the same dose (300 mg). Mean plasma levels of GLPG1972 were similar in healthy, fed Japanese and White individuals following multiple daily doses of 300 mg. Increased urinary 6β‐OH‐cortisol/cortisol ratios in 5 of 18 healthy volunteers after multiple daily dosing suggests potential mild CYP3A4 induction. Pharmacokinetic parameters were overall comparable across the populations studied, confirming the robustness and reliability of these data.

In the analysis of pharmacodynamic effects, plasma/serum ARGS levels were significantly and consistently reduced in the 3 studies following repeated administration of GLPG1972. In study B, levels reached a floor after ≈2 weeks, where they remained at steady state until the last dose of GLPG1972 on day 29. ARGS levels returned to baseline levels following cessation of GLPG1972 treatment, suggesting the interaction between GLPG1972 and ADAMTS‐5 is reversible. Based on these results and the limited incremental ARGS reductions observed at doses higher than 300 mg in studies A and C, it is reasonable to assume that a plateau effect would have been achieved in studies A and C if dosing had continued beyond 14 days. This pharmacodynamic analysis, therefore, supports a consistent treatment‐related reduction in plasma/serum ARGS levels in healthy volunteers and participants with OA, and further confirms the target engagement of the molecule. These observations also support the previous human explant study in which GLPG1972 dose‐dependently inhibited production of aggrecanase‐derived aggrecan fragments.^15^ However, how this translates to a clinical meaningful effect on cartilage thickness and/or pain and function remains to be determined.

Findings from the 3 studies also support the safety and tolerability of GLPG1972 in healthy adult men (of both White and Japanese origin) and in male and female participants with OA. Only 2 participants experienced serious AEs, few TEAEs led to study drug discontinuation (those that did were either protocol related or resolved upon study drug discontinuation), and no deaths occurred in any of the studies. The majority of TEAEs were rated mild in severity, and headache was the most frequently reported TEAE. No clinically relevant abnormalities in ECGs were observed. Across all 3 studies, administration of single (up to 2100 mg) and multiple (up to 1050 mg daily) ascending oral doses of GLPG1972 was generally well tolerated.

In terms of potential study limitations, it should be noted that study A used a solution of GLPG1972 for oral administration, whereas GLPG1972 was administered in tablet form in studies B and C. However, no major differences in pharmacokinetic profiles were observed between the solution and the tablet forms of GLPG1972. It should also be noted that although treatment‐related reductions in ARGS provide confidence that GLPG1972 engages with the target ADAMTS‐5 in participants with OA, these studies were not designed to demonstrate clinical efficacy. Finally, the relatively limited number of included participants across the studies suggests caution is needed with regard to interpreting the observed overall generally favorable safety profile of GLPG1972.

## Conclusions

The pharmacokinetic profile of the potential OA medication GLPG1972 is described in a series of randomized, double‐blind, placebo‐controlled phase 1 studies in healthy participants and participants with OA. GLPG1972 was rapidly absorbed, and plasma exposure was proportional to dose in fasted healthy participants. Following multiple daily dosing in fed volunteers, steady state was achieved after 2 days of dosing, and accumulation was minimal. In healthy volunteers and participants with OA, concentrations of aggrecan ARGS in serum or plasma decreased over a period of 2 weeks of daily dosing in a dose‐dependent manner, indicative of engagement of GLPG1972 with the target ADAMTS‐5. GLPG1972 was generally well tolerated following single and multiple oral doses. The efficacy and safety of GLPG1972 has been investigated in a phase 2 clinical study in participants with knee OA.^24^

## Funding

These studies were funded by Galapagos and Servier.

## Conflicts of Interest

E.v.d.A., H.D., and A.F. are employees of and hold stock options in Galapagos NV. S.D. and P.P. are employees of and hold stock options in Galapagos SASU. L.S.L. has received personal fees from Arthro Therapeutics AB, AstraZeneca, Paradigm, Pfizer, and Regeneron outside of the submitted work. A.L. and E.L. are employees of the Institut de Recherches Internationales Servier. D.A. is an employee of and holds stock options in Galapagos SASU and is named on patent WO2016102347 issued to Galapagos. S.L. and A.S. declare no conflicts of interest.

## Data‐Sharing Statement

All data relevant to the study are included in the article or supplemental information; additional data will not be made available.

## Supporting information

Supporting Information

Supporting Information

Supporting Information
