# Supplementary material for: Safety, Pharmacokinetics, and Pharmacodynamics of the ADAMTS‐5 Inhibitor GLPG1972/S201086 in Healthy Volunteers and Participants With Osteoarthritis of the Knee or Hip
Source: Clin Pharmacol Drug Dev. 2021 Dec 2;11(1):112–22. doi: 10.1002/cpdd.1042 (PMC9299907; doi:10.1002/cpdd.1042)
Supplement: Supplementary file 1 — Supporting Information [file CPDD-11-112-s003.docx]

**Supporting Material**


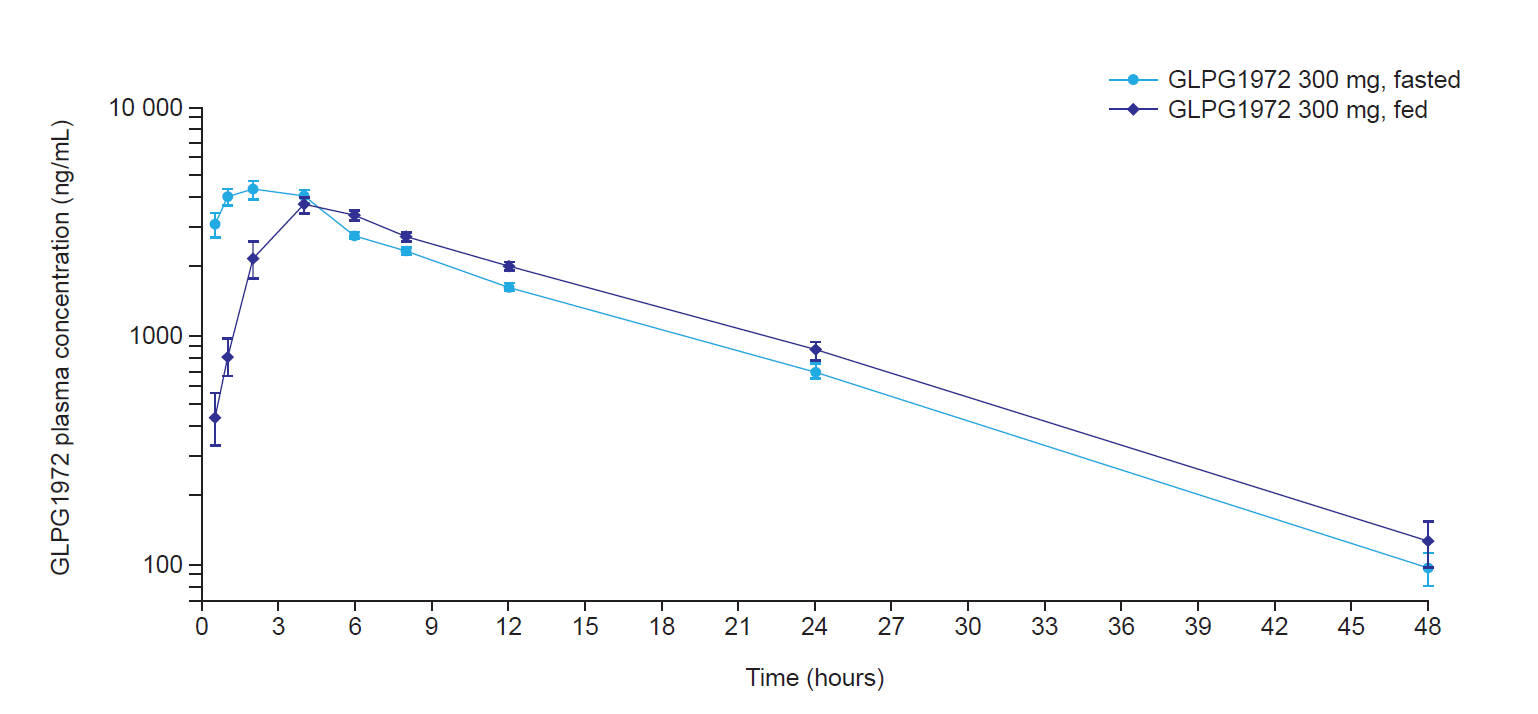


**Supplemental Figure 1**. GLPG1972 plasma concentrations over time in fasted and fed healthy participants following a single dose of GLPG1972.

Data obtained from Study A. Data show mean ± standard error.


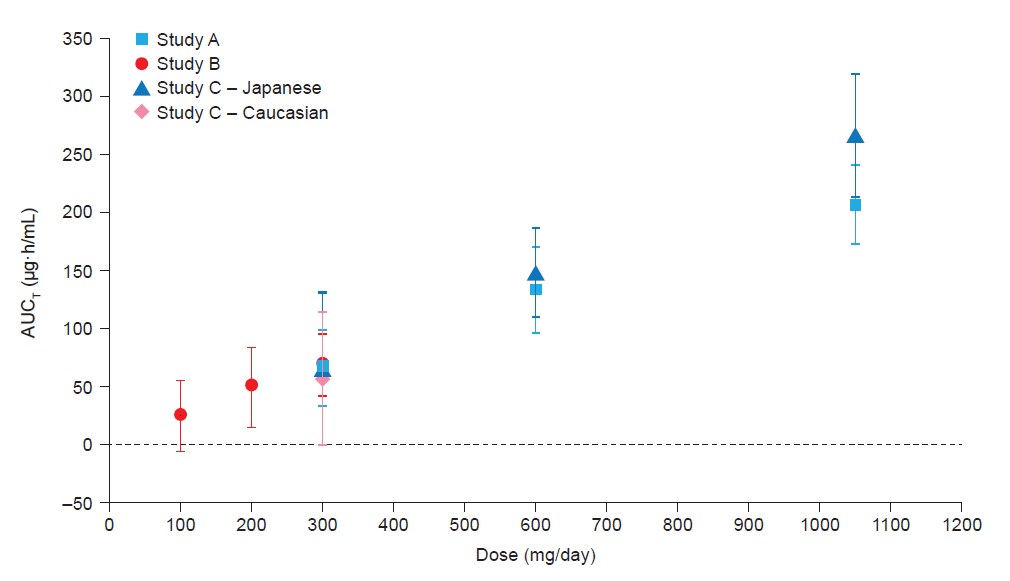


**Supplemental Figure 2.** Area under the plasma concentration–time curve over the dose-interval following the last dose of GLPG1972.

Data show MAD phase day 14 (Study A and C) or day 15 (Study B) mean AUC_T_ ± %CV.

AUC_T_, area under the plasma concentration–time curve over the dosing interval; %CV, coefficient of variation; MAD, multiple ascending doses.

**Supplemental Table S1.** Demographic Characteristics of Study Participants

Study A (first in human; healthy participants)

|  | Single ascending dose phase | | | Multiple ascending dose phase | | | | |
| --- | --- | --- | --- | --- | --- | --- | --- | --- |
|  | Cohort A (n = 9) | Cohort B (n = 8) | Total SAD (n = 17) | Pooled placebo (n = 6) | GLPG1972 300 mg/day (Cohort C) (n = 6) | GLPG1972 600 mg/day (Cohort D) (n = 6) | GLPG1972 1050 mg/day (Cohort E) (n = 6) | Total MAD   (n = 24) |
| Age, years, mean (SE)  Median (range) | 30.1 (1.94)  29.0 (21-38) | 35.6 (3.55)  36.5 (20-49) | 32.7 (2.01)  30.0 (20-49) | 42.8 (2.43)  42.5  (34-50) | 41.2 (4.32)  43.5 (21-50) | 43.3 (3.12)  45.5 (32-50) | 36.8 (3.44)  38.0 (24-48) | 41.0 (1.67)  42.0 (21-50) |
| Height, cm, mean (SE)  Median (range) | 183.3 (1.39)  183.5 (175.0-189.0) | 178.4 (2.56)  178.1  (168.0-186.5) | 181.0 (1.49)  183.4 (168.0-189.0) | 182.2 (2.62)  180.5 (175.0-194.0) | 176.5 (2.83)  175.8 (167.2-187.5) | 176.5 (1.49)  178.0 (171.0-180.0) | 176.7 (1.34)  176.3 (173.5-182.0) | 178.0 (1.13)  178.0 (167.2-194.0) |
| Weight, kg, mean (SE)  Median (range) | 77.2 (3.25)  80.8 (62.9-89.7) | 76.8 (4.96)  74.2 (61.9-104.0) | 77.0 (2.81)  74.3 (61.9-104.0) | 82.2 (3.87)  83.6 (64.4-90.1) | 85.5 (3.54)  88.0 (69.1-94.3) | 79.1 (2.76)  78.8 (71.1-88.9) | 81.4 (2.64)  82.6 (71.5-89.3) | 82.0 (1.59)  84.0 (64.4-94.3) |
| BMI, kg/m^2^_,_ mean (SE)  Median (range) | 23.0 (1.0)  23.5 (19.6-29.3) | 24.0 (1.1)  22.6 (21.3-30.0) | 23.5 (0.7)  22.8 (19.6-30.0) | 24.8 (1.2)  25.3 (19.7-27.8) | 27.4 (0.7)  28.3 (24.7-28.9) | 25.4 (0.8)  25.7 (22.1-28.1) | 26.1 (0.9)  26.4 (23.2-28.4) | 25.9 (0.5)  26.1 (19.7-28.9) |
| White, n (%) | 9 (100.0) | 7 (87.5) | 16 (94.1) | 6 (100.0) | 4 (66.7) | 5 (83.3) | 6 (100.0) | 21 (87.5) |
| Male, n (%) | 9 (100.0) | 8 (100.0) | 17 (100.0) | 6 (100.0) | 6 (100.0) | 6 (100.0) | 6 (100.0) | 24 (100.0) |

Study B (participants with OA)

| Parameter | Pooled placebo (n = 6) | GLPG1972 100 mg (n = 8) | GLPG1972 200 mg (n = 8) | GLPG1972 300 mg (n = 8) | Total (n = 30) |
| --- | --- | --- | --- | --- | --- |
| Age, years, mean (SE)  Median (range) | 61.8 (3.15)  62.5 (52-70) | 61.6 (2.74)  61.0 (50-72) | 62.3 (1.54)  60.0 (52-71) | 61.1 (2.22)  63.5 (56-67) | 61.7 (1.14)  61.5 (50-72) |
| Height, cm, mean (SE)  Median (range) | 168.0 (1.88)  168.0 (162-174) | 171.3 (4.33)  170.5 (156-187) | 170.0 (3.82)  168.0 (162-177) | 168.6 (1.73)  166.0 (155-189) | 169.6 (1.58)  168.0 (155-189) |
| Weight, kg, mean (SE)  Median (range) | 78.2 (4.82)  75.0 (66.7-101.2) | 83.5 (6.30)  91.6 (55.4-103.5) | 76.7 (4.87)  72.8 (58.5-93.4) | 74.5 (4.35)  74.9 (59.3-104.1) | 78.2 (2.55)  75.0 (55.4-104.1) |
| BMI, kg/m^2^_,_ mean (SE)  Median (range) | 27.7 (1.5)  27.4 (22.5-33.4) | 28.1 (1.1)  28.4 (22.8-33.1) | 26.4 (0.8)  26.5 (21.0-33.1) | 26.2 (1.4)  26.6 (21.8-29.1) | 27.1 (0.6)  27.2 (21.0-33.4) |
| Race, n (%)  Black or African American  White | 2 (33.3) 4 (66.7) | 2 (25.0) 6 (75.0) | 1 (12.5) 7 (87.5) | 1 (12.5) 7 (87.5) | 6 (20.0) 24 (80.0) |
| Sex, n (%)  Male  Female | 4 (66.7) 2 (33.3) | 3 (37.5) 5 (62.5) | 1 (12.5) 7 (87.5) | 4 (50.0) 4 (50.0) | 12 (40.0) 18 (60.0) |

Study C (Japanese and Caucasian)

| A) Single ascending dose phase | | | | | | | | | | | |
| --- | --- | --- | --- | --- | --- | --- | --- | --- | --- | --- | --- |
|  | Japanese participants | | | | | | | | Caucasian participants | | |
|  | Placebo (n = 12) | GLPG1972 50 mg (n = 6) | GLPG1972 150 mg (n = 6) | GLPG1972 300 mg (n = 6) | GLPG1972 600 mg (n = 6) | GLPG1972 1050 mg (n = 6) | GLPG1972 1500 mg (n = 6) | Total  (n = 48) | Placebo  (n = 2) | GLPG1972 300 mg (n = 6) | Total  (n = 8) |
| Age (years), mean (SD) | 28.5 (5.1) | 23.8 (5.0) | 23.2 (3.4) | 24.3 (2.3) | 27.2 (4.7) | 23.5 (3.3) | 25.3 (4.7) | 25.5 (4.6) | 28.0 (7.1) | 29.8 (4.2) | 29.4 (4.5) |
| Weight (kg), mean (SD) | 66.4 (7.6) | 67.1 (4.3) | 63.8 (5.5) | 61.1 (4.2) | 64.8 (10.4) | 65.6 (4.6) | 62.6 (6.2) | 64.7 (6.5) | 71.7 (10.9) | 68.9 (4.2) | 69.6 (5.6) |
| Height (cm), mean (SD) | 171.4 (5.6) | 173.5 (5.5) | 171.7 (7.1) | 168.8 (4.2) | 173.5 (5.4) | 172.2 (2.9) | 172.7 (4.8) | 171.9 (5.1) | 178.0 (8.5) | 180.3 (5.5) | 179.8 (5.8) |
| BMI (kg/m^2^), mean (SD) | 22.7 (2.2) | 22.4 (1.5) | 21.9 (1.4) | 21.6 (2.1) | 21.4 (2.7) | 22.0 (2.2) | 21.1 (1.8) | 22.0 (2.0) | 22.9 (1.0) | 21.5 (0.7) | 21.8 (1.0) |

| B) Multiple ascending dose phase | | | | | | | | |
| --- | --- | --- | --- | --- | --- | --- | --- | --- |
|  | Japanese participants | | | | | Caucasian participants | | |
|  | Placebo (n = 6) | GLPG1972 300 mg (n = 6) | GLPG1972 600 mg (n = 6) | GLPG1972 1050 mg  (n = 6) | Total (n = 24) | Placebo (n = 2) | GLPG1972 300 mg (n = 6) | Total (n = 8) |
| Age (years), mean (SD) | 27.7 (6.3) | 28.8 (5.1) | 24.7 (4.4) | 27.5 (3.3) | 27.2 (4.8) | 27.5 (3.5) | 30.5 (7.1) | 29.8 (6.3) |
| Weight (kg), mean (SD) | 59.9 (2.4) | 63.4 (4.4) | 62.2 (12.1) | 62.8 (3.5) | 62.1 (6.4) | 68.9 (16.4) | 75.1 (10.7) | 73.5 (11.4) |
| Height (cm), mean (SD) | 173.2 (3.1) | 172.5 (5.2) | 172.2 (8.1) | 173.3 (3.4) | 172.8 (5.0) | 173.5 (21.9) | 178.8 (6.7) | 177.5 (10.4) |
| BMI (kg/m^2^), mean (SD) | 20.1 (0.9) | 21.3 (2.0) | 20.8 (2.5) | 21.0 (0.9) | 20.8 (1.7) | 23.1 (0.4) | 23.8 (3.0) | 23.6 (2.6) |

BMI, body mass index; SD, standard deviation.

**Supplemental Table S2.** Mean 6β-OH-Cortisol to Cortisol Ratio in Urine in the MAD phase of Study A

|  | GLPG1972 300 mg  (n = 6) | GLPG1972 600 mg  (n = 6) | GLPG1972 1050 mg  (n = 6) |
| --- | --- | --- | --- |
| Day −1 mean (%CV) | 7.67 (60.2) | 7.64 (52.0) | 11.0 (58.1) |
| Day 13 mean (%CV) | 13.6 (18.1) | 7.80 (26.8) | 16.7 (29.4) |
| Day 13/ Day −1 mean (%CV) | 1.77 (75.6) | 1.02 (44.0) | 1.52 (84.8) |

CV, coefficient of variation; MAD, multiple ascending dose

**Supplemental Table S3.** GLPG1972 Plasma Pharmacokinetics Observed in Healthy Fasting Participants in the SAD Phase of Study C

|  | GLPG1972 dose | | | | | | |
| --- | --- | --- | --- | --- | --- | --- | --- |
|  | 50 mg (n = 6) | 150 mg (n = 6) | 300 mg (n = 6) | 300 mg (n = 6) | 600 mg (n = 6) | 1050 mg (n = 6) | 1500 mg (n = 6) |
| Parameter | Japanese | Japanese | Japanese | Caucasian | Japanese | Japanese | Japanese |
| C_max_ (mean [CV%], μg/mL) | 0.58 (9.5) | 1.87 (21) | 2.87 (38) | 2.83 (27) | 4.94 (11) | 6.29 (17) | 10.78 (9.6) |
| t_max_ (median [range], h) | 4.0 (4.0-4.0) | 4.0 (4.0-4.0) | 4.0 (2.0-4.0) | 4.0 (4.0-6.0) | 4.0 (4.0-6.0) | 4.0 (1.0-4.0) | 3.0 (2.0-4.0) |
| AUC_0-∞_ (mean [CV%], μg·h/mL) | 7.98 (17) | 27.69 (27)^a^ | 45.17 (44)^a^ | 51.04 (23)^a^ | 78.04 (13)^a^ | 116.79 (11)^b^ | 151.76 (44)^a^ |
| t_1/2,z_ (mean [CV%], h) | 9.6 (32) | 12 (41) | 13 (88) | 16 (46) | 12 (67) | 20 (43) | 14 (74) |

AUC, area under the plasma concentration–time curve; C_max_, maximum observed plasma concentration; SAD, single ascending dose; t_1/2,z_, terminal elimination half-life; t_max_, time of occurrence of C_max_.

^a^n = 5. ^b^n = 4.

**Supplemental Table S4.** GLPG1972 Plasma Pharmacokinetics in Healthy Fed Participants in the MAD Phase of Study C

|  |  | Dose GLPG1972 | | | |
| --- | --- | --- | --- | --- | --- |
|  |  | 300 mg (n = 6) | 300 mg (n = 6) | 600 mg (n = 6) | 1050 mg (n = 6) |
| Day | Parameter | Japanese | Caucasian | Japanese | Japanese |
| Day 1 | C_max,D1_ (mean [CV%], μg/mL) | 4.72 (13) | 4.08 (24) | 8.72 (28) | 15.92 (10) |
|  | t_max,D1_ (median [range], h) | 4.0 (4.0-6.0) | 4.0 (4.0-4.0) | 4.0 (2.0-6.0) | 4.0 (4.0-6.0) |
|  | AUC_0-24,D1_ (mean [CV%] μg·h/mL) | 50.70 (5.3) | 43.65 (28) | 107.05 (35) | 205.73 (13) |
|  | t_1/2,z,D1_ (mean [CV%], h) | 7.8 (14) | 8.4 (18) | 9.5 (32) | 11 (15) |
| Day 14 | C_max,D14_ (mean [CV%], μg/mL) | 5.56 (16) | 5.08 (25) | 12.02 (24) | 18.78 (9.3)^a^ |
|  | t_max,D14_ (median [range], h) | 4.0 (2.0-4.0) | 4.0 (4.0-4.0) | 4.0 (4.0-4.0) | 4.0 (4.0-4.0)^a^ |
|  | AUC_τ_ (mean [CV%], μg·h/mL) | 65.06 (7.4) | 56.77 (27) | 147.82 (27) | 265.95 (20)^a^ |
|  | t_1/2,z,D14_ (mean [CV%], h) | 8.7 (16) | 9.0 (17) | 9.8 (25) | 10 (28)^a^ |

AUC_0-24_, area under the plasma concentration–time curve from time 0 to 24 hours post dose; AUC_T_, area under the plasma concentration–time curve over the dosing interval (i.e. 24 hours post dose); C_max_, maximum observed plasma concentration; D1, day 1; D14, day 14; t_1/2,z_, terminal elimination half-life; t_max_, time of occurrence of C_max_

^a^n = 4, as two participants withdrew.

**Supplemental Table S5.** Plasma Accumulation Ratios of GLPG1972 in Healthy Fed Participants in the MAD Phase of Study C

|  | Dose GLPG1972 | | | |
| --- | --- | --- | --- | --- |
|  | 300 mg (n = 6) | 300 mg (n = 6) | 600 mg (n = 6) | 1050 mg (n = 4)^a^ |
| Parameter | Japanese | Caucasian | Japanese | Japanese |
| R_ac(Cmax)_ (mean [CV%]) | 1.2 (11) | 1.2 (11) | 1.4 (6.8) | 1.2 (11) |
| R_ac(AUCτ)_ (mean [CV%]) | 1.3 (7.5) | 1.3 (4.9) | 1.4 (14) | 1.3 (17) |

AUC_T_, area under the plasma concentration–time curve over the dosing interval (i.e. 24 hours post dose); C_max_, maximum observed plasma concentration; MAD, multiple ascending doses; R_ac_, accumulation ratio.

^a^Two participants withdrew.

R_ac(Cmax)_ was calculated by Day 14 C_max_ divided by Day 1 C_max_

R_ac(AUCτ)_ was calculated by Day 14 AUCτ divided by Day1 AUCτ

**Supplemental material 1**

The high-fat, high-calorie breakfast had the following composition: 2 eggs fried in butter, 2 slices of bacon, 2 slices of toast with butter, 113 g of hash brown potatoes (fried potatoes), 240 mL of whole milk. A high-fat (about 50% of total calorie content) and high-calorie (approximately 800 to 1000 calories) meal derives about 150, 250 and 500–600 calories from protein, carbohydrate and fat, respectively.^1^

An example of a standardized breakfast is: 4 slices of bread, 2 slices of ham and/or cheese, butter, jelly, glass of water (150 mL). Approximately 533 calories.

An example of a standardized evening meal is: turkey fillet, mashed potatoes, vegetables, glass of water (150 mL). Approximately 553 calories.

1. FDA Guidance for Industry, Food-Effect Bioavailability and Fed Bioequivalence Studies, December 2002.

**Supplemental material 2**

GLPG1972 concentrations in plasma were determined by using a validated LC/MS-MS method. Prior to injection into the analytical system, isolation of GLPG1972 and its stable isotope labelled interna standard (G962581 – deuterated GLPG1972) from 20µL of human Li-heparin plasma was performed by protein precipitation with 150µL of acetonitrile. The protein precipitated mixture was centrifuged at 3500 RPM for approximately 5 minutes at +4°C. Then, 60µL of the clear supernatant was transferred in a 96-well plate and 60µL of water was added. The sealed plate was then vortex mixed at 1400 RPM for approximately 3 minutes. 5µL to 20µL of the reconstituted sample was injected into the chromatographic system. Chromatographic separation was performed on an Acquity BEH C18 column (50 x 2.1 mm, 1.7 µm from Waters) set at 40°C by using an Acquity UPLC system (Waters) working in a gradient elution mode. The aqueous mobile phase consisted of a 0.1% formic acid in acetonitrile (mobile phase A) and of a 0.1% formic acid in water mixture (mobile phase B). Either a TSQ Vantage mass spectrometer (Thermo) or an API5000 mass spectrometer (AB Sciex) equipped with an electrospray probe operated in the multiple reaction monitoring (MRM) in positive mode was used for quantification. The precursor‐to‐product ion pairs at the mass‐to‐charge ratio (m/z) were 407 to 213 and 411 to 217 for GLPG1972 and G962581, respectively for the TSQ vantage system. When the API5000 system was used the MRM transitions were 407 to 123 and 411 to 123 for GLPG1972 and G962581, respectively. The calibration curves in plasma were linear over the range of 1–1000 ng/mL with 1/x2 as weighting factor. The limit of quantification of the assay in plasma was set at 1 ng/mL.

Cortisol and 6β-hydroxycortisol concentrations in urine were determined using a validated LC/MS-MS method. Prior to injection into the analytical system, isolation of both cortisol and 6β-hydroxycortisol with their stable isotope labelled internal standards (cortisol-d4 and 6-β-hydroxycortisol-d4) from human urine was performed by solid phase extraction (Oasis HBL 30mg 96-well extraction plate, Waters). Then, the eluates were evaporated and the residue reconstituted with the injection solvent before injection into the chromatographic system. Chromatographic separation was performed on an Kinetex C18 (100 x 3.0 mm, 2.6 µm from Phenomenex) by using a 1290 Infinity UPLC system (Agilent) working in a gradient elution mode. The aqueous mobile phase consisted of a mixture of high purity water/acetonitrile containing 2 mM of ammonium acetate and 0.1% of formic acid. An API4000 mass spectrometer (AB Sciex) equipped with an electrospray probe operated in the multiple reaction monitoring (MRM) in positive mode was used for quantification. The precursor‐to‐product ion pairs at the mass‐to‐charge ratio (m/z) were 363 to 121 and 367 to 121 for cortisol and cortisol-d4, respectively. For the 6β-hydroxycortisol and the 6-β-hydroxycortisol-d4 the MRM transitions were 379 to 325 and 383 to 329, respectively. The calibration curves in urine were linear over the range of 1–500 ng/mL for the cortisol and over the range 2-1000 ng/mL for the 6β-hydroxycortisol concentrations using 1/x2 as weighting factor. The limit of quantification of the assay in urine was set at 1 ng/mL and 2 ng/mL for cortisol and 6β-hydroxycortisol, respectively.
