# Supplementary figures and images for: Safety, Pharmacokinetics, and Pharmacodynamics of the ADAMTS‐5 Inhibitor GLPG1972/S201086 in Healthy Volunteers and Participants With Osteoarthritis of the Knee or Hip
Source: Clin Pharmacol Drug Dev. 2021 Dec 2;11(1):112–22. doi: 10.1002/cpdd.1042 (PMC9299907; doi:10.1002/cpdd.1042)

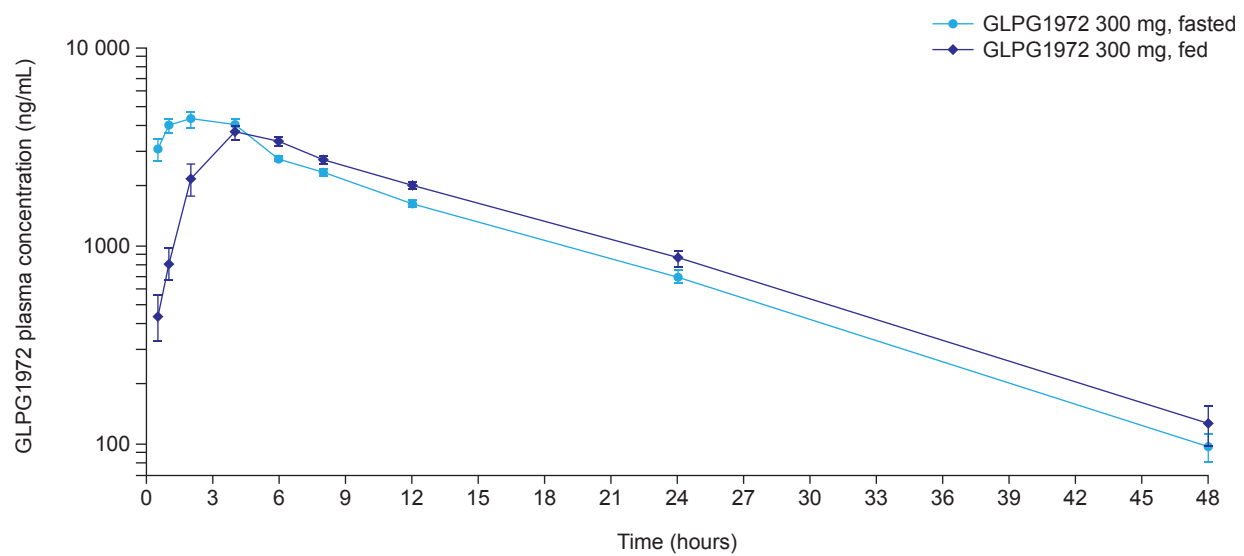

Supplement: Supplementary file 2 — Supporting Information [file CPDD-11-112-s001.pdf]

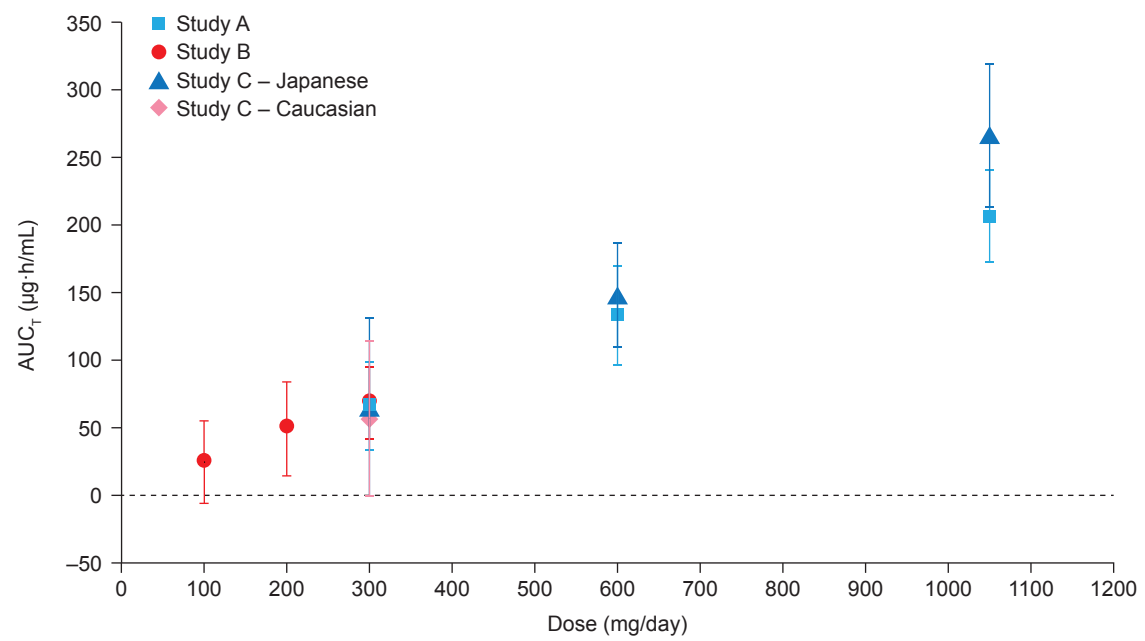

Supplement: Supplementary file 3 — Supporting Information [file CPDD-11-112-s002.pdf]
